# Supplementary material for: Sleep-dependent memory consolidation in infants protects new episodic memories from existing semantic memories
Source: Nat Commun. 2020 Mar 10;11:1298. doi: 10.1038/s41467-020-14850-8 (PMC7064567; doi:10.1038/s41467-020-14850-8)
Supplement: Supplementary file 1 — Supplementary Information [file 41467_2020_14850_MOESM1_ESM.pdf]

Sleep-dependent memory consolidation in infants protects new episodic memories from existing semantic memories

Manuela Friedrich, Matthias Mölle, Angela D. Friederici, and Jan Born

SUPPLEMENTARY INFORMATION

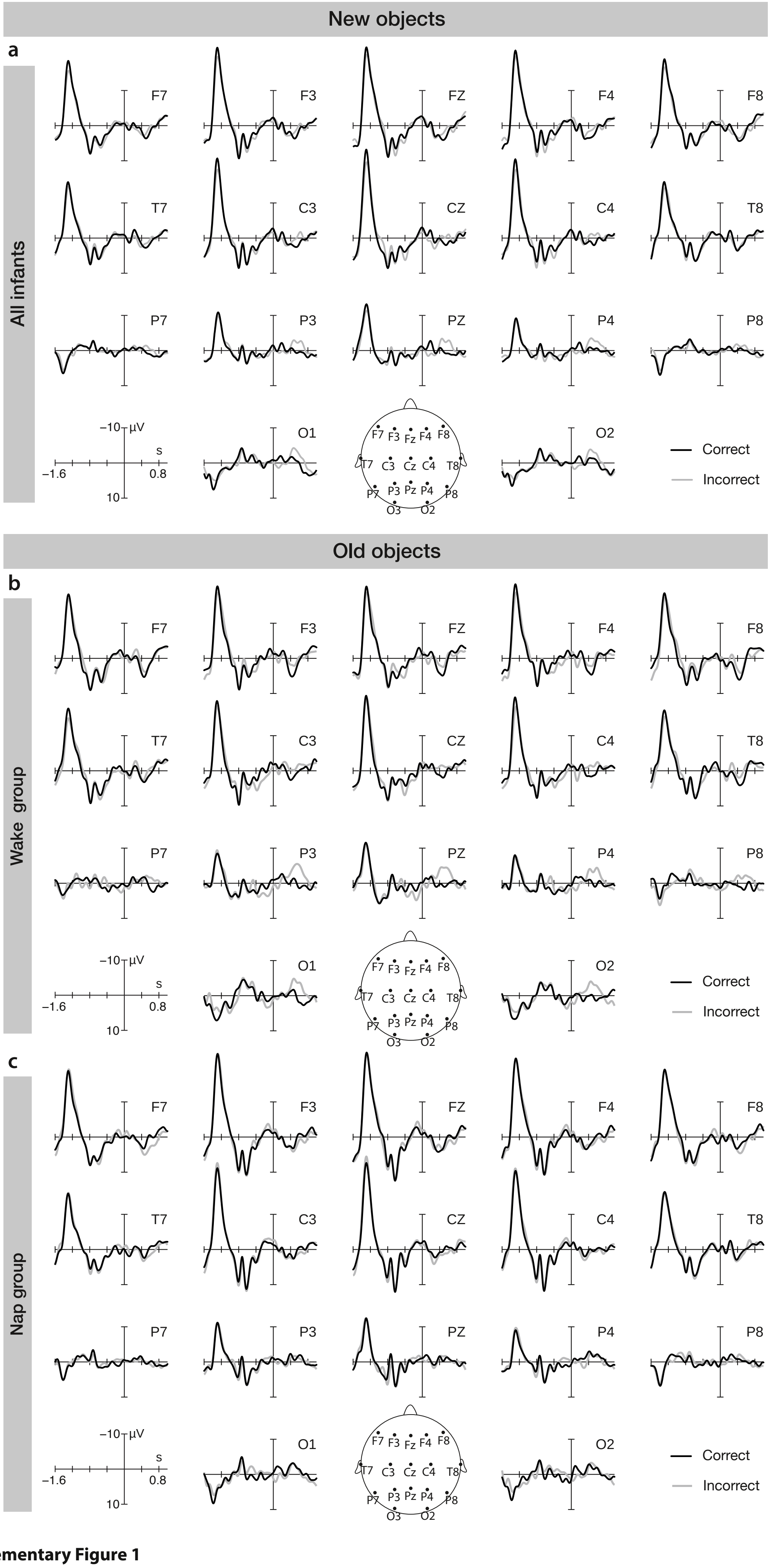

Supplementary Figure 1
N400 semantic memory effect and FTMR episodic memory effect. The Figure is related to Figure 2 of the main manuscript and shows the ERP responses at individual electrode sites. Source data are provided as a Source Data file.

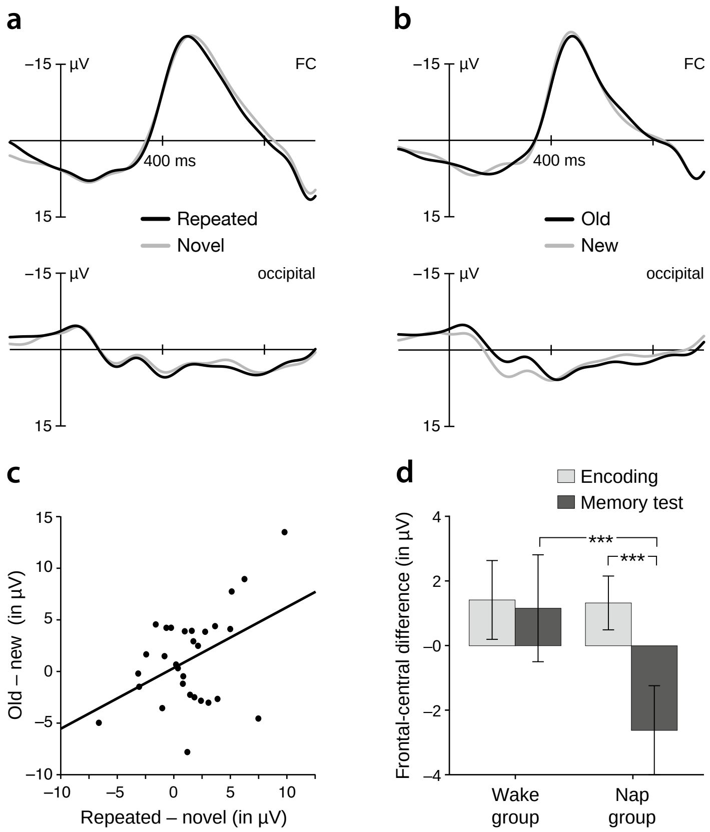

Supplementary Figure 2

Immediate memory and later memory for the individual objects.

a ERP responses of the overall group to repeated (black lines) and novel (grey lines) objects time-locked to picture onset at encoding. Late object repetition effect (600 – 800 ms) indicating immediate object memory.

b ERP responses of the overall group to old (black lines) and new (grey lines) objects at the memory test. Early occipital old/new object memory effect (150 – 300 ms). Missing late object memory effect in the overall group due to the polarity-inversed ERP responses in the wake and nap groups.

c Correlation between the late frontal-central object repetition effect during encoding and the non-significant late frontal-central old/new object recognition difference in the memory test of the wake group ($r = .434$, $P = .016$).

d Mean late frontal-central ERP differences (±2 s.e.m.) of the wake and nap groups at encoding (repeated – novel) and at the memory test (old – new). No difference between nap and wake groups at encoding ($t_{58} = -.126$, $P = .900$). No significant change from encoding to memory test in the wake group ($t_{29} = .327$, $P = .746$). Significant change from encoding to memory test in the nap group ($t_{29} = 4.520$, $P = .0001$, $d = -.825$) and significant difference between nap group and wake group in the memory test ($t_{58} = -3.506$, $P = .001$, $d = -.829$), both indicating strong modifications of infant object memory during the post-encoding nap.

Source data are provided as a Source Data file.
