# Supplementary material for: Sleep-dependent memory consolidation in infants protects new episodic memories from existing semantic memories
Source: Nat Commun. 2020 Mar 10;11:1298. doi: 10.1038/s41467-020-14850-8 (PMC7064567; doi:10.1038/s41467-020-14850-8)
Supplement: Supplementary file 3 — Reporting Summary [file 41467_2020_14850_MOESM3_ESM.pdf]

nature research

Corresponding author(s): NCOMMS-19-08391-C

Last updated by author(s): 19.11.2019

Reporting Summary

Nature Research wishes to improve the reproducibility of the work that we publish. This form provides structure for consistency and transparency in reporting. For further information on Nature Research policies, see Authors & Referees and the Editorial Policy Checklist .

Statistics

For all statistical analyses, confirm that the following items are present in the figure legend, table legend, main text, or Methods section.

n/a	Confirmed	
☐	☒	The exact sample size (n) for each experimental group/condition, given as a discrete number and unit of measurement
☐	☒	A statement on whether measurements were taken from distinct samples or whether the same sample was measured repeatedly
☐	☒	The statistical test(s) used AND whether they are one- or two-sided<br>*Only common tests should be described solely by name; describe more complex techniques in the Methods section.*
☒	☐	A description of all covariates tested
☐	☒	A description of any assumptions or corrections, such as tests of normality and adjustment for multiple comparisons
☐	☒	A full description of the statistical parameters including central tendency (e.g. means) or other basic estimates (e.g. regression coefficient) AND variation (e.g. standard deviation) or associated estimates of uncertainty (e.g. confidence intervals)
☐	☒	For null hypothesis testing, the test statistic (e.g. F, t, r) with confidence intervals, effect sizes, degrees of freedom and P value noted<br>*Give P values as exact values whenever suitable.*
☒	☐	For Bayesian analysis, information on the choice of priors and Markov chain Monte Carlo settings
☒	☐	For hierarchical and complex designs, identification of the appropriate level for tests and full reporting of outcomes
☐	☒	Estimates of effect sizes (e.g. Cohen's d, Pearson's r), indicating how they were calculated

Our web collection on statistics for biologists contains articles on many of the points above.

Software and code

Policy information about availability of computer code

Data collection	- Presentation: ERTS (BeriSoft)<br>- EEG data: QREFA-Software (Twente Medical Systems International)<br>- Sleep data: DOMINO Steuerung (Somnomedics)
Data analysis	- EEP software package, MPI for Human Cognitive and Brain Sciences, Leipzig, commercially available as EEProbe, ANT Neuro, Germany<br>- Brain Vision Analyzer, Brain Products GmbH, Germany<br>- REMBRANDT Diagnostic software / Polysomnography, Natus Medical, Pleasanton, USA<br>- SPIKE2 Cambridge Electronic Design, Milton, Cambridge, ENGLAND<br>- SPSS Statistics 22 IBM, Armonk, USA

For manuscripts utilizing custom algorithms or software that are central to the research but not yet described in published literature, software must be made available to editors/reviewers. We strongly encourage code deposition in a community repository (e.g. GitHub). See the Nature Research guidelines for submitting code & software for further information.

Data

Policy information about availability of data

All manuscripts must include a data availability statement. This statement should provide the following information, where applicable:

- Accession codes, unique identifiers, or web links for publicly available datasets
- A list of figures that have associated raw data
- A description of any restrictions on data availability

All the relevant data is available upon reasonable request. Inquiries should be directed to the corresponding author. Source data are provided as a Source Data file.

Field-specific reporting

Please select the one below that is the best fit for your research. If you are not sure, read the appropriate sections before making your selection.

☒ Life sciences ☐ Behavioural & social sciences ☐ Ecological, evolutionary & environmental sciences

For a reference copy of the document with all sections, see nature.com/documents/nr-reporting-summary-flat.pdf

Life sciences study design

All studies must disclose on these points even when the disclosure is negative.

Sample size	Sample size was determined based on previous infant studies.
Data exclusions	An additional 47 infants were measured, but excluded from the analyses because of too few artefact-free trials and very noisy ERP responses (N = 34), because infants allocated to the nap group did not fall asleep after the encoding session (N = 5), because of fussiness, crying, or missing attention in one of the experimental sessions (N = 4), or due to technical problems with the acquisition of the sleep EEG (N = 4).
Replication	*Describe the measures taken to verify the reproducibility of the experimental findings. If all attempts at replication were successful, confirm this OR if there are any findings that were not replicated or cannot be reproduced, note this and describe why.*
Randomization	Experimental groups were recruited consecutively.
Blinding	Blinding was not possible.

Reporting for specific materials, systems and methods

We require information from authors about some types of materials, experimental systems and methods used in many studies. Here, indicate whether each material, system or method listed is relevant to your study. If you are not sure if a list item applies to your research, read the appropriate section before selecting a response.

Materials & experimental systems

n/a	Involved in the study	
☒	☐	Antibodies
☒	☐	Eukaryotic cell lines
☒	☐	Palaeontology
☒	☐	Animals and other organisms
☐	☒	Human research participants
☒	☐	Clinical data

Methods

n/a	Involved in the study	
☒	☐	ChIP-seq
☒	☐	Flow cytometry
☒	☐	MRI-based neuroimaging

Human research participants

Policy information about studies involving human research participants

Population characteristics	60 infants (26 female, mean age 15 months and 11 days, sd 30 days)<br>All infants were born in the 36th to 41st week of pregnancy with a birth weight ranging from 2100 g to 4700 g (3512 ± 520 g). They had no known visual or hearing deficits and no major sleep problems. All infants were habitual nappers. Prior to the lab visit, infants were assigned to either the wake group or the nap group. The nap group (N = 30, 11 female) and the wake group (N = 30, 15 female) did not significantly differ in age (T_{59} = –1.561, P = .124), gestational age at birth (T_{54} = –.164, P = .871), birth weight (T_{54} = .076, P = .940), and Apgar score (median of the APGAR score at ten minutes after birth: 10 in both groups, Kolmogorov-Smirnov-Z = –.815, P = .415).
Recruitment	Participants were recruited via the local residents' registration office.
Ethics oversight	We have complied with all relevant ethical regulations. The study was approved by the ethics committee of the Humboldt University of Berlin.

Note that full information on the approval of the study protocol must also be provided in the manuscript.
